# The Trials and Tribulations of Assembling Large Medical Imaging Datasets for Machine Learning Applications

**DOI:** 10.1007/s10278-021-00505-7

**Published:** 2021-10-04

**Authors:** Kirti Magudia, Christopher P. Bridge, Katherine P. Andriole, Michael H. Rosenthal

**Affiliations:** 1 Department of Radiology, Brigham & Women’s Hospital, Boston, MA USA; 2 Present Address: Department of Radiology, Duke University School of Medicine, Durham, NC 27710 USA; 3 MGH & BWH Center for Clinical Data Science, Boston, MA USA

**Keywords:** Exam retrieval, Artificial intelligence, Dataset, Informatics

## Abstract

With vast interest in machine learning applications, more investigators are proposing to assemble large datasets for machine learning applications. We aim to delineate multiple possible roadblocks to exam retrieval that may present themselves and lead to significant time delays. This HIPAA-compliant, institutional review board–approved, retrospective clinical study required identification and retrieval of all outpatient and emergency patients undergoing abdominal and pelvic computed tomography (CT) at three affiliated hospitals in the year 2012. If a patient had multiple abdominal CT exams, the first exam was selected for retrieval (*n*=23,186). Our experience in attempting to retrieve 23,186 abdominal CT exams yielded 22,852 valid CT abdomen/pelvis exams and identified four major categories of challenges when retrieving large datasets: cohort selection and processing, retrieving DICOM exam files from PACS, data storage, and non-recoverable failures. The retrieval took 3 months of project time and at minimum 300 person-hours of time between the primary investigator (a radiologist), a data scientist, and a software engineer. Exam selection and retrieval may take significantly longer than planned. We share our experience so that other investigators can anticipate and plan for these challenges. We also hope to help institutions better understand the demands that may be placed on their infrastructure by large-scale medical imaging machine learning projects.

## Background

Machine learning is a field focusing on how computers can learn from data and sits at the intersection between statistics and computer science. An increasingly popular approach to machine learning is to use deep neural networks, inspired by the structure and function of the human brain to process complex image data [1]. Indeed, such networks are now commonplace in many industries for tasks such as recognizing faces. In radiology, machine learning has the potential to improve the speed and accuracy of the radiologist’s workflow [2].

A major bottleneck to the potential progress of machine learning in radiology is the assembly of imaging datasets to use for model training [3]. Performance of these models generally improves with more data so maximal dataset size is desired [1]. There are examples of efforts to assemble large public datasets—datasets easily accessible for research that can be downloaded from public websites or require acceptance of nonburdensome data use agreements to download data—with the hope of spurring innovation [4–8]. However, public data, which must be stripped of identifying data to protect the privacy of the source subjects under HIPAA and IRB guidelines, is not available for all possible clinical applications and may not generalize to “real world” data, which exists in and is acquired from clinical systems as would be encountered in a routine clinical setting [9–12]. Thus, increasing numbers of investigators are proposing to assemble their own datasets for training, validating, and testing machine learning models for research and development purposes.

While retrieving exams from radiology systems initially appears to be a simple step in a machine learning project, many roadblocks may present themselves, leading to significant time delays in gathering the number of exams desired for a given project. Our aim was to retrieve a single outpatient CT abdomen/pelvis exam for each patient imaged in a multiple hospital system in 2012, in total 23,186 exams, and delineate all possible retrieval-related issues that researchers may face.

## Methods

Following Mass General Brigham (formerly Partners Healthcare) institutional review board approval and with HIPAA-compliant study procedures, all patients that underwent an outpatient CT abdomen/pelvis at Brigham & Women’s Hospital, Massachusetts General Hospital or Dana-Farber Cancer Institute in 2012 were identified by the Mass General Brigham Healthcare Research Patient Data Registry. The data provided by this registry included all radiology exams for outpatients that had at least one CT abdomen/pelvis exam in 2012 (1.7 million exams). This data was then limited to all CT exams; to patients imaged in the year 2012; to exam descriptions of “Abd”; group of exam not “chest,” “hdnk” (head and neck), “unclassified,” “resp” (respiratory), “lextr” (lower extremity), or “cspin” (cervical spine); to type of patient not “inpatient”; and age between 18 and 99; resulting in 33,182 exams for 23,186 unique patients. We selected the earliest exam for each of the included patients to limit our dataset to a single exam per adult outpatient that underwent abdominal CT in 2012, in total 23,186 exams.

## Results

Four major categories of challenges when retrieving large datasets were identified: cohort selection and processing, retrieving DICOM exam files from PACS, data storage, and non-recoverable failures (see Table 1).

**Table 1:** Summary of challenges encountered during exam retrieval

	Problem	Solution	Result
Cohort selection and processing	Mislabeled exams *(i.e interventional and musculoskeletal labeled as abdominal CTs)*	Excluded exam descriptions of “ablation, fna, biopsy, drainage, guidance, drain, drg, bx, interventional, interv, perc, bone”	22,903 exams remaining
	Inconsistent formatting of medical record numbers (MRNs) and accessions (ACCs)	For one hospital, MRNs were padded with leading zeros to 8 digits. All ACCs before a change in EMR had a leading “A” removed.	MRN and ACCs for 10,089 exams were reformatted
	Some ACCs generated solely for billing with no linked images *(i.e. CT abdomen/pelvis with separate ACCs for abdomen, pelvis, and contrast)*	Queried copies of the underlying databases of both hospital clinical PACS systems with MRN and date to identify all candidate ACCs CTs with >20 images Search for exam with body part of abdomen If none, allow body part of “pelvis” or “chest” If none, expand date range to +/- 4 days	838 exams had different ACC chosen than what was provided from the research database
	Inconsistent linkage of images to ACCs *(i.e. images could be either with the abdomen or pelvis ACCs)*		
Exam retrieval	Slow pull method for one hospital consisted of a Web API pull method that preceded a vendor-neutral archive	New method established where radiology IT pushes exams to a DCM4CHEE instance (an open-source DICOM image management system), which is then transferred to our storage.	Original time estimate to retrieve exams of >1 year. With new method, exams retrieved in 2 weeks
	Push rate from DCM4CHEE instance exceeding write rate to storage, causing crashes	Slowed down push rate and added memory to the server running the DCM4CHEE instance to buffer images as they came in before they were written to storage	No further system crashes during exam retrieval
Data storage	Data storage requirement exceeded available storage in a multi-user system	Transitioned project files to new storage device	Overall delay of 3 weeks
Non-recoverable failures	MRN/ACC discrepancies	Excluded from further analysis	17 exams
	Topogram only exam		7 exams
	Missing exam		8 exams
	Corrupted CT data		3 exams
	Non-patient test exam		1 exam
	DICOM encoding errors		15 exams
**Final number of exams**			**22,852 exams**

### Cohort Selection and Processing

An initial attempt to retrieve our cohort revealed that a number of studies that we had identified for retrieval were actually mislabeled musculoskeletal and interventional CT exams. In order to identify these incorrectly included exams, we excluded exams with exam descriptions containing “ablation,” “fna,” “biopsy,” “drainage,” “guidance,” “drain,” “drg,” “bx,” “interventional,” “interv,” “perc,” or “bone.” This led to the exclusion of 283 of the original 23,186 exams selected.

Another major initial challenge for exam retrieval was inconsistent formatting of medical record numbers (MRNs) and accession numbers (ACCs) across different hospitals. At one hospital, the MRNs provided by the research database originally identifying our cohort had leading zeroes that were dropped during export. Furthermore, at this hospital, the research database added a leading “A” to all ACCs before a change in electronic medical record systems. These formatting changes were not consistent with our radiology information system that interfaced with PACS. In total, we reformatted the MRNs and ACCs for 10,089 exams.

Our next roadblock related to how multiple body part exams are handled by the radiology information systems. First, we found that some ACCs were generated solely for billing with no linked images. For instance, a CT abdomen/pelvis could have separate ACCs for the abdomen, pelvis, and contrast. Furthermore, there was inconsistent linkage of images to these separate ACCs such that the images were most often linked to the abdomen ACC, but they could also be linked to the pelvis ACC, or even the chest ACC if chest, abdomen, and pelvis CT exams were acquired together. These linkages varied over time due to changing systems and policies.

To address these frequent inconsistencies, we accessed copies of the underlying databases of both hospital clinical PACS systems and attempted to identify the correct ACC *de novo* by querying with the patient’s MRN and date of exam. Candidate ACCs were first limited to those ACCs corresponding to CT exams with greater than 20 CT slices. This was performed to exclude empty ACCs and frequently occurring partial imports of outside scans. Next, the remaining ACCs were limited to exams with body parts of “abdomen, GI, GU or body.” However, if no ACCs met those requirements, body parts of “pelvis” and if necessary “chest” were permitted to account for the times that the pelvis and chest accessions were linked to the images for the abdomen/pelvis CT exam. If no ACCs met the criteria above, the acceptable date range for ACCs meeting the criteria was expanded to four days before and after the exam date provided by the research database. We often found that exam dates were inconsistently reported in the research database and variably related to the actual exam date, PACS receipt date, or report signing date; for example, exams performed close to midnight in the emergency room may be assigned to the following day. In total, 838 exams had a different ACC chosen than what was originally provided by the research database. Of note, an ACC not containing the abdominal CT images of interest was still selected by the logic described above for 24 exams, which were manually corrected.

### Exam Retrieval

Our original exam retrieval method for one hospital consisted of a website-based application programming interface (API) that preceded a vendor-neutral archive. This method had been sufficient for small prior projects; however, the estimated time for exam retrieval for our proposed project using this system was greater than one year. Part of the contribution to the extended time estimate was that exam retrieval was limited to nights to avoid slowing down the clinical performance of PACS. However, this time estimate did not include allowances for accommodating other competing exam retrieval requests.

To improve the speed of exam retrieval, a new method was established whereby the hospital Radiology information technology department pushed exams to an open-source image archive (DCM4CHEE) which was then transferred to the exam storage system. Initially, the exam retrieval was unstable with this new method. Investigation revealed that the push rate from the DCM4CHEE instance exceeded the write rate to storage. This was resolved by slowing down the push rate and adding memory to the server running the DCM4CHEE instance to buffer images as they came in before they were written to storage. With this new method and the described modifications, all exams were retrieved within 2 weeks.

### Data Storage

The PACS systems associated with the multiple hospitals in our hospital system did not provide a mechanism to estimate the on-disk storage requirement until the data are exported. Thus, sufficient storage could not be accurately planned for in advance of retrieval. Unfortunately, in the middle of exam retrieval, we exceeded the available storage in a multi-user shared network storage device. A new storage device was brought online, and the project files were transitioned to this device. This led to an overall delay of 3 weeks.

### Non-recoverable Failures

With careful investigation of exams that failed retrieval, a number of non-recoverable failures were identified and excluded from future analysis. In total, 51 exams were excluded from further analysis due to discrepancies between the MRN and ACC pairs found in the patient data registry and the PACS, exams with only topograms, missing exams, corrupted CT data, non-patient test exams, and DICOM encoding errors.

### Project Time

Troubleshooting the issues highlighted above took 3 months and at a minimum of 300 hours of time between the primary investigator (a radiologist), a data scientist, and a software engineer.

## Discussion

Our experience demonstrates that while automating image analysis using machine learning has the potential to enable imaging-based research at unprecedented scales, selecting and retrieving the required radiology exams presents a considerable logistical task that may require considerable time and manual intervention. While the complexity of these steps is often initially overlooked, they are likely to consume a considerable fraction of the time and resources for the project and it is vitally important to budget sufficiently for them. Challenges faced during the assembly of large datasets for machine learning may include cohort selection and processing, retrieving DICOM exam files from PACS, data storage, and non-recoverable failures.

Evolving database policies, for one, can become a major obstacle to efficient data science research. This project was significantly delayed by several historical changes in the system of accession numbers that were never brought under a consistent policy. The solution to this problem required participation by people with institutional memories that spanned these changes and would have been difficult to identify and address without that support. Many of the obstacles that are described in this study would have been mitigated by enforcement of consistent exam identification and dating during prior system migration steps. The choice to defer difficult data migration steps, while expedient in the near term, can impose significant long-term costs that may serve as a barrier to research and clinical applications of artificial intelligence.

The mechanism of exam retrieval itself may present a major challenge. At one hospital in our study, the retrieval infrastructure was not robust enough to support retrieving the vast number of exams necessary in a reasonable time frame. We were able to increase retrieval speed by a factor of 100 through a software solution, but further improvements required a major change in the hardware configuration for the enterprise. Exam retrieval can be limited by hardware, software, and security decisions, so institutions that wish to facilitate future data science research should actively include data science requirements in their infrastructure planning process.

Some institutions are beginning to mitigate retrieval mechanism concerns by purposefully designing research access into their image storage and retrieval architectures. It is crucial that researchers have a role in drafting requirements and specifications for these systems if they will be used to support research. If research exam retrievals directly compete with retrievals for clinical work, then robust systems should be in place to ensure prioritization of exam retrievals for clinical workflow. Institutions can design access mechanisms that do not directly compete, but this requires thoughtful and deliberate design.

Planning for adequate research data storage is a critical consideration when assembling a large medical imaging dataset for machine learning. It may be difficult to estimate the storage required given that most retrieval systems and PACS likely will not provide an estimate for the required storage in advance of the retrieval. Many data storage options are also typically part of a multi-user system, adding to the complexity of predicting whether sufficient data storage will be available. Furthermore, there is a tradeoff between exam retrieval rate and research storage demands. High retrieval rate systems may not need large research stores if studies can be retrieved dynamically, whereas slow rate systems may need very large archives to hold data during extended research efforts.

It should also be expected that a certain small percentage of exams will not be able to be retrieved as a result of unresolvable database discrepancies and data integrity failures. While varying by institution and type of study, researchers should anticipate a 1–5% rate of loss from their initial cohort.

Our study was limited to the experience of a single project at a single multi-hospital system. However, many other projects at our hospital system have faced similar challenges. We believe that the themes identified in this study will generalize to other institutions and perhaps motivate common data standards for clinical imaging data, including for exam descriptions *such as RadLex/Logical Observation Identifiers Names and Codes (LOINC) *[9]*.*

In conclusion, cohort selection and assembly may take significantly longer than planned for machine learning projects in medical imaging. Challenges range from cohort selection and processing, retrieving DICOM exam files from PACS, data storage, and non-recoverable failures. We share our experience so that other investigators can anticipate and plan for these potential roadblocks to save valuable project time and resources. We also hope to help institutions better understand the demands that may be placed on their infrastructure by large-scale medical imaging machine learning projects.

## Data Availability

Possible by request to the corresponding author on a case-by-case basis.

## Declarations

The authors declare that they had full access to this manuscript and take complete responsibility for the integrity and the accuracy of the submitted manuscript. 
